# Implementation of patient education at first and second dispensing of statins in Dutch community pharmacies: the sequel of a cluster randomized trial

**DOI:** 10.1186/1472-6963-11-313

**Published:** 2011-11-16

**Authors:** Caroline HPA Van de Steeg-van Gompel, Michel Wensing, Peter AGM De Smet

**Affiliations:** 1 Scientific Institute for Quality of Healthcare (IQ healthcare), Radboud University Nijmegen Medical Centre, PO Box 9101, 6500 HB Nijmegen, The Netherlands; 2 Department of Clinical Pharmacy, Radboud University Nijmegen Medical Centre, Nijmegen, The Netherlands; 3 Scientific Institute of Dutch Pharmacists, The Hague, The Netherlands

## Abstract

**Background:**

As a result of the previous part of this trial, many patients with cardiovascular disease were expected to receive a statin for the first time. In order to provide these patients with comprehensive information on statins, as recommended by professional guidance, education at first and second dispensing of statins had to be implemented. This study was designed to assess the effectiveness of an intensive implementation program targeted at pharmacy project assistants on the frequency of providing education at first dispensing (EAFD) and education at second dispensing (EASD) of statins in community pharmacies.

**Methods:**

The participating community pharmacies were clustered on the basis of local collaboration, were numbered by a research assistant and subsequently an independent statistician performed a block randomization, in which the cluster size (number of pharmacies in each cluster) was balanced. The pharmacies in the control group received a written manual on the implementation of EAFD and EASD; the pharmacies in the intervention group received intensive support for the implementation. The impact of the intensive implementation program on the implementation process and on the primary outcomes was examined in a random coefficient logistic regression model, which took into account that patients were grouped within pharmacy clusters.

**Results:**

Of the 37 pharmacies in the intervention group, 17 pharmacies (50%) provided EAFD and 12 pharmacies (35.3%) provided EASD compared to 14 pharmacies (45.2%, *P *= 0.715) and 12 pharmacies (38.7%, *P *= 0.899), respectively, of the 34 pharmacies in the control group. In the intervention group a total of 72 of 469 new statin users (15.4%) received education and 49 of 393 patients with a second statin prescription (12.5%) compared to 78 of 402 new users (19.4%, *P *= 0.944) and 35 of 342 patients with a second prescription (10.2%, *P *= 0.579) in the control group.

**Conclusion:**

The intensive implementation program did not increase the frequency of providing EAFD and EASD of statins in community pharmacies.

**Trial Registration:**

clinicaltrials.gov NCT00509717

## Background

In October 2006 we started a trial (NCT00509717) in order to improve the prescribing of statins for patients with CVD in general practice [1]. As a result of this trial, many patients with CVD were expected to receive a statin for the first time. In order to provide these patients with comprehensive information on statins, as recommended by professional guidance, education at first and second dispensing of statins had to be implemented.

Patient education is a basic element of community pharmacy practice according to the Dutch Pharmacy Standard [2] and according to several international guidelines [3]. Such patient education is required from a legal and ethical perspective, and it is also recommended to ensure that patients understand how to use medication safely and effectively [3]. At the time this study was designed, providing education at first and second dispensing of medication in community pharmacies was also expected to improve medication adherence and persistence in the Netherlands, although evidence for that was scarce. Poor medication adherence or even total discontinuation of statin therapy for secondary prevention is highly prevalent and an important problem because it increases mortality and cardiovascular events [4,5]. Use of statins for secondary prevention rapidly decreases in the first months, with a gradually smaller decrease on the longer term [6]. Between 60% and 86% of the patients still use a statin one year after their first cardiovascular event [7,8], and between 44% and 80% after two or more years [6,9-11]. So, enhancing adherence and persistence with statin therapy is important for optimal secondary prevention of CVD for the patients in our trial.

Patient education is not provided by all pharmacies to all patients at each dispensing. A review from 36 studies in the USA, Europe, Australia and Canada shows that counseling was provided by 50% to 100% of the pharmacies to 8% to 80% of the patients [3]. The barriers for implementing patient education in community pharmacy which we identified from the first author's own experience as a pharmacist, from pharmacists' reported barriers and facilitators in a previous trial in which we implemented another pharmaceutical care service [12] and from the literature [13,14] were: A) Organizational: making time for introducing patient education to the pharmacy technicians and for educating them; lack of time and space for providing patient education; patients' privacy; the availability of protocols for patient education; lack of documentation of patient education that was provided; lack of reimbursement; involvement of pharmacy technicians in the implementation process; B) With regard to the individual professionals: knowledge and skills of pharmacists and pharmacy technicians; attitude towards patient education; C) Factors concerning the social context: patient indifference.

Successful implementation of patient education requires a comprehensive approach, which targets relevant barriers and facilitators to change in a specific setting [15]. Unfortunately, an evidence-based strategy to translate identified barriers to a tailor-made implementation intervention is still lacking [16]. So, after having identified the potential barriers for the implementation of patient education, we used common sense to design the intensive implementation intervention, thus following the planning model described by Grol and Wensing [17]. Our intensive multifaceted implementation program was directed at pharmacists and at pharmacy technicians who were appointed as project assistant. This program consisted of an educational manual, two interactive educational meetings, reminder and feedback telephone calls and reminder newsletters.

The aim of the present study was to test the hypothesis that the intensive implementation program would increase the frequency of patient education provided at first and second dispensing of statins in community pharmacies.

## Methods

A cluster randomized trial was conducted between September 2006 and February 2008. The medical ethical committee Arnhem-Nijmegen gave approval for this trial. The trial consisted of two parts: in the first part, which is described elsewhere [1], prescribing of statins for secondary prevention of cardiovascular disease was targeted. In the second part, which is described here, the focus was on patient education at the moment patients presented a statin prescription in a community pharmacy for the first or second time.

### Participants

Community pharmacies were recruited through a mailing to all 211 pharmacies in the South of the Netherlands in April 2006. Participation was voluntary, though encouraged by one of the two major health insurance companies in this part of the Netherlands: pharmacies participating in this study were exempted from presenting an annual plan and from reporting of patient care activities. There was no financial incentive for participation in the project other than the usual dispensing fee per prescription.

Patients who were selected in the first part of the trial (which were patients who already used antiplatelet medication but not a statin) and who received a first or a second statin prescription were eligible for education by a pharmacist or pharmacy technician.

### Randomization

In order to ensure that pharmacies in the control group would not be influenced by the intensive implementation program, all pharmacies that cooperated with the same GPs or that had local collaboration with another pharmacy in the vicinity were grouped in a cluster of pharmacies. The clustered pharmacies were numbered by a research assistant. An independent statistician performed a block randomization, in which the cluster size (number of pharmacies in each cluster) was balanced.

### Interventions

In all pharmacies, both in the control group and in the experimental group, pharmacists and pharmacy technicians were instructed to provide education at first dispensing (EAFD) and education at second dispensing (EASD) to new statin users, guided by the same protocols for EAFD and EASD. The difference between the two groups consisted of the way they were supported with the implementation of EAFD and EASD.

### Directed at patients

The protocol for EAFD stated that the pharmacist or pharmacy technician should read out loud the patient label including information on the name, strength and use of the statin, and should explain the importance of long-term use of the statin to each patient. If the patient appeared to be interested and if there was enough time, one or more of the following issues could be discussed as well: indication of the statin; how it works; side effects; what to do when a dose intake was missed; interactions; and if everything was clear to the patient or whether he or she had any more questions. We advised the pharmacies not to give too much information at first dispensing, because patients generally receive a lot of information from the physician at the visit in which they receive their first statin prescription. The information provided in the pharmacy should be sufficient to motivate the patient to use the statin at least until the second dispensing, which is the moment at which the patient's experiences with using the medication are discussed and more information can be provided.

According to the protocol, EASD (which is normally two weeks after first dispensing in The Netherlands) started with the question how the patient had experienced statin use. When the patient had a positive experience, pharmacists and pharmacy technicians were instructed to emphasize the importance of long-term use once more and to mention the possibility to ask questions about medications in the pharmacy. When the patient expressed a negative experience, pharmacists and pharmacy technicians were instructed to find out whether the patient had any specific questions or problems and whether they could help the patient with that.

### Directed at pharmacies: experimental group

Experimental pharmacies received a written educational manual in September or October 2006, which consisted of information about the project; step-by-step instructions for carrying out the project; protocols for EAFD and EASD, an explanation of these protocols and examples of possible situations at second dispensing for the pharmacy technicians to practice with; and a flow-chart for the planning of the project. The project had a planned duration of 12 months. Pharmacists and pharmacy technicians who were appointed as project assistant were also invited to an interactive educational meeting, which was based on the literature [14] and on the extensive experience of two professional communication advisors who were well-informed about daily practice in community pharmacies. After an explanation of the project (what, why, how, by whom, when) by one of the researchers (CvdS), pharmacists and project assistants split up. Pharmacists mainly discussed the first part of the trial; the project assistants discussed their experience with EAFD and EASD, and expectations with regard to this project under the guidance of two research assistants and guided by a questionnaire. In a second interactive educational meeting, project assistants were challenged by a communication advisor to identify facilitators of implementation and to identify and tackle potential barriers for implementation with regard to the content of the protocols; organization of patient education; and knowledge and motivation of the pharmacy technicians. In the four reminder telephone calls which were planned with each pharmacist for the first part of the trial, the project assistants were also interviewed and supported with the implementation of patient education.

### Directed at pharmacies: control group

The control group pharmacies received a written educational manual in October 2006, which was the same as the manual for the experimental pharmacies. No further intervention was applied.

### Measurements

For each time education was given at first and second dispensing, the pharmacist or pharmacy technician marked the items which were discussed on a written checklist. "EAFD as recommended" included both having read out loud the patient label with information on the name, strength and use of the statin; and having explained the importance of long-term use. Statin prescriptions for patients who received at least one antiplatelet drug as well as sex, age and prescriptions for other cardiovascular drugs or drugs with a statin interaction were extracted from a national prescription database, which consists of all dispensing data from more than 1,670 of the 1,850 community pharmacies in the Netherlands. The pharmacies which provide data to this database serve about 13.5 million people, and dispense drugs or medical aids some 140 million times per year (more information at http://www.sfk.nl/algemeen/english.html). A statin prescription was considered a first prescription if there were no statin prescriptions in the database in the six months before the pharmacy started the first part of the trial. Follow-up data on statin use were available until February 2008. Pharmacy characteristics were measured at the start of the study by means of a written inventory. Barriers and facilitators with regard to the implementation process in the experimental pharmacies were collected by means of the telephone interviews which were part of the intensive implementation program.

### Outcomes

The primary outcome of the study was the percentage of patients with a first prescription for a statin who received EAFD. The secondary outcome of the study was the percentage of patients with a second prescription for a statin who received EASD. Additional outcomes were perceived barriers and facilitators to implementation in the intervention group and the effect of EAFD and EASD on persistence with statin therapy.

### Sample size

Because this was the sequel of a trial which was designed to enhance statin prescribing for patients with cardiovascular disease, the sample size needed was not calculated for the primary outcome of this study, but for the primary outcome of the first part of the trial. We planned to include 7980 patients from 76 pharmacies.

### Statistical analysis

The impact of the intensive implementation program on the implementation process and on the primary outcomes was examined in a random coefficient logistic regression model, which took into account that patients were grouped within pharmacy clusters. The primary analysis was quasi intention-to-treat [18] and involved all patients of whom statin dispensing data were known. *P*-values of 0.05 or less were considered significant. Determinants for providing EAFD were tested for using unpaired t-tests and Chi-squared tests. Data were analyzed using the SPSS 16.0 software package, except the random coefficient logistic regression analysis, which was performed using the SAS 9.1.3 software by means of the Glimmix procedure.

## Results

### Participating pharmacies, loss to follow-up and data completeness

**Figure 1:**
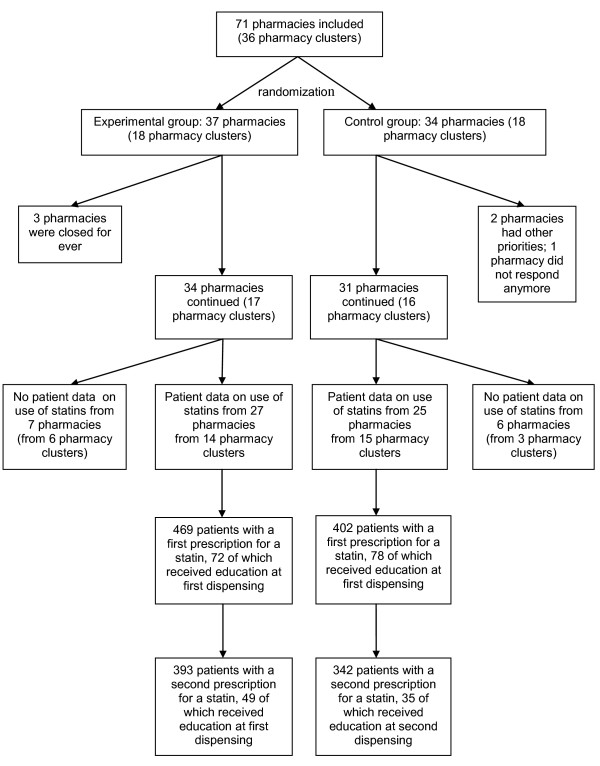
**Participant flow**.

The flow of participants throughout the study is shown in Figure 1. In total, 71 eligible pharmacies registered for participation in the study between May and August 2006, which were grouped into 36 clusters (taking into account local collaborations). Randomization resulted in a control group of 34 pharmacies in 18 pharmacy clusters and an experimental group of 37 pharmacies in 18 pharmacy clusters.

Six pharmacies withdrew between September and November 2006: three of them were closed for ever; two had changed priorities; and one pharmacy did not respond to repeated attempts to obtain information. Patient data at baseline and follow-up were available for 52 pharmacies: one pharmacy did not give authorization to the national database administrator for data extraction; one pharmacy had opened in October 2005 and did not have 12 complete months of patient data before the start of the project, which was required for the first part of the trial; and for 11 pharmacies data extraction was impossible because patient identification numbers changed during the project due to a switch of the pharmacy to another computer system.

In the 52 pharmacies for which patient data were available, 871 of the selected patients were eligible for EAFD since they received a first statin prescription within 12 months after the start of the project and 735 patients also received a second statin prescription, which made them eligible for EASD as well.

### Success of the implementation

The indicators for success of the implementation are shown in Table 1. In the control group, 14 pharmacies (45.2%) did provide EAFD and 12 pharmacies (38.7%) did provide EASD, respectively, versus 17 pharmacies (50.0%, *P *= 0.715) and 12 pharmacies (35.3%, *P *= 0.899), respectively, in the experimental group. There were no differences between the two groups in the primary or in the secondary outcome: 78 patients (19.4%) with a first statin prescription received EAFD and 35 patients (10.2%) received EASD in the control group, versus 72 patients (15.5%, *P *= 0.944) and 49 patients (12.5%, *P *= 0.579) in the experimental group. Also, the percentage of new users who received EAFD in the pharmacies which did implement EAFD was similar in both groups. The pharmacy which performed best, offered EAFD and EASD to 15 of the 19 patients (78.9%) who received a first statin prescription after the pharmacy had started providing EAFD. The Intracluster correlation coefficient (ICC) for the primary outcome was 0.347. In the pharmacies which did provide EASD to at least one patient, a higher percentage of the new statin users received EASD in the experimental group, but this difference did not reach significance: 28.2% versus 17.1% in the control group (*P *= 0.172) in the multilevel analysis.

**Table 1:** Indicators for success of implementation

	Experimental group	Control group	OR (95% CI)	*P*-value
Frequency of EAFD offered (% of first prescriptions) ^a^	72/469 (15.4)	78/402 (19.4)	0.96 (0.31;3.01)	0.944

Number of pharmacies which offered EAFD (%)	17/34 (50.0)	14/31 (45.2)	1.24 (0.38;4.00)	0.715

Frequency of EAFD offered in the pharmacies which performed EAFD (% of first prescriptions) ^b^	72/252 (28.6)	78/257 (30.4)	1.08 (0.51;2.29)	0.845

Frequency of EASD offered (% of second prescriptions) ^a^	49/393 (12.5)	35/342 (10.2)	1.41(0.42;4.78)	0.579

Number of pharmacies which offered EASD (%)	12/34 (35.3)	12/31 (38.7)	0.93 (0.27;3.20)	0.899

Frequency of EASD offered in the pharmacies which did perform EASD (% of second prescriptions) ^c^	49/174 (28.2)	35/205 (17.1)	1.85 (0.76;4.50)	0.172

EAFD, education at first dispensing; EASD, education at second dispensing; OR, odds ratio

^a ^Data from the pharmacies of which the total number of first statin prescriptions was known, which were 25 pharmacies in the control group and 27 pharmacies in the experimental group.

^b ^Data from the pharmacies which did perform EAFD and of which the total number of first statin prescriptions was known: 12 pharmacies in the control group and 13 pharmacies in the experimental group.

^c ^Data from the pharmacies which did perform EASD and of which the total number of second statin prescriptions was known: 10 pharmacies in the control group and 10 pharmacies in the experimental group.

Table 2 shows the content of education in subjects who received education at first dispensing. The patient label was read out loud at first dispensing more frequently in the experimental group than in the control group (86.1% versus 69.2%, *P *= 0.017), and more patients received EAFD on the indication for the statin in the experimental group (77.8% versus 59.0%, *P *= 0.016).

**Table 2:** Content of education in patients or patient representatives who received education at first dispensing

	Experimental group (*N *= 72)	Control group (*N *= 78)	*P*-value
Patient himself/herself fetched medication and was interested	49 (68.1)	47 (60.3)	0.323

Patient label read out loud ^a;b^	62 (86.1)	54 (69.2)	0.017

Importance of long-term use ^a^	53 (73.6)	47 (60.3)	0.098

Indication of the statin	56 (77.8)	46 (59.0)	0.016

How statin works	44 (61.1)	45 (57.7)	0.661

Side effects	47 (65.3)	48 (61.5)	0.636

What to do when a dose intake is missed	20 (27.8)	24 (30.8)	0.577

Interactions	20 (27.8)	27 (34.6)	0.352

Contact for more information	33 (45.8)	36 (46.2)	0.931

At least one of these	65 (90.3)	55 (70.5)	0.006

Both subjects which were recommended in protocol EAFD	51 (70.8)	47 (60.3)	0.179

EAFD, education at first dispensing

Data are absolute frequencies, with the percentage between brackets.

^a ^Recommended in the protocol for education at first dispensing

^b ^The patient label includes information on the name, strength and use of the statin.

In Table 3 the pharmacy characteristics related to education at first dispensing are shown (see also, Appendix). None of the pharmacy characteristics was significantly related to providing EAFD.

**Table 3:** Characteristics of pharmacies related to education at first dispensing

	Pharmacies which did educate patients at first dispensing (*N *= 31^a^)	Pharmacies which did not educate patients at first dispensing (*N *= 34)	*P*-value
Number of years in service pharmacist ^b^	16.5 ± 9.0	14.5 ± 8.5	0.382

Frequency of postgraduate training in pharmacotherapy for pharmacist: Regularly or Often	28 (90.3)	32 (94.1)	0.566

Attitude regarding care-providing function (continuous scale of 16-80) ^b;c^	66.2 ± 6.6	66.3 ± 6.6	0.969

Pharmacy technicians with specialized care-providing duties	18 (58.1)	23 (67.6)	0.424

Relationship with GP: Good or very good	22 (71.0)	19 (55.9)	0.208

Workload as perceived by pharmacist: High or very high	10 (32.3)	10 (29.4)	0.804

Pharmacy is part of chain or franchise formula	21 (67.7)	22 (64.7)	0.796

Participation in study because of perception of pressure from insurance company: Partly or completely agree	5 (16.1)	6 (17.6)	0.870

Number of first statin prescriptions within 6 months of start of the project	11.7 ± 8.0	7.9 ± 7.3	0.082

Team was used to providing education at first dispensing of (some types of) medication	30 (96.8)	30 (88.2)	0.197

GP, general practitioner

Data are absolute scores (% in brackets) unless otherwise specified

^a ^17 pharmacies from the experimental group and 14 pharmacies from the control group

^b ^Data are mean scores ± SD

^c ^Attitude was measured using the statements which are described in the Appendix

### Barriers and facilitators

The barriers and facilitators for implementing education at first and second dispensing, reported by the project assistants in the experimental group, are shown in Table 4. The main barrier was that the pharmacist chose (either explicitly or implicitly) not to start with EAFD and EASD: In three pharmacies the project had discontinued completely, in five pharmacies the pharmacist did not want to start before the general practitioners had reviewed and returned the list with selected patients on which they had marked the patients with a statin indication, which was late or did not happen at all. In five other pharmacies, the pharmacist did not take much initiative to retrieve the lists or to start with EAFD and EASD, according to the project assistants. The project assistant and other pharmacy technicians were discouraged by that. Other barriers with regard to the implementation were insufficient staff (5×); negative reactions from patients (4×); lack of routine because of the limited number of new statin users (2×); lack of experience with EASD (2×); forgetting to fill in the checklist sometimes (2×); other priorities in the pharmacy (1×); and difficulties with opening a conversation at the moment of second dispensing (1×).

**Table 4:** Barriers and facilitators for the implementation of EAFD and EASD reported by project assistants of experimental pharmacies

Barriers related to the:	Influenced which step of implementation process	Frequency of reporting (number of pharmacies)
Individual professionals		
Lack of pharmacist's initiative to start with EAFD and EASD	Getting started	8
Pharmacist wanted to wait for list returned from GPs	Getting started	5
Forgetting to fill in the checklist sometimes	Registration	2
Difficulties with opening a conversation at the moment of second dispensing	Getting started and continuation	1

Organizational context		
Insufficient staff	Getting started	5
Lack of routine because of limited number of new statin users	Continuation	2
Lack of experience with EASD	Getting started	2
Other priorities in pharmacy	Getting started	1

Patients		
Negative reactions of patients	Continuation	4

Facilitators related to the:		

Individual professionals		
Enthusiastic pharmacy technicians	Getting started	8

Organizational context		
Pharmacy technicians used to providing EAFD and/or EASD	Getting started	7
Availability of project materials	Getting started	2
Computer reminder at the moment of first or second dispensing	Continuation	2
Patients were informed already by GP	Continuation	2
Feedback to pharmacy technicians by pharmacist	Continuation	1
Materials on a visible place	Continuation	1
EAFD and EASD fits well into regular work system	Continuation	1

Patients		
Positive reactions of patients	Continuation	5

Perceived facilitators were enthusiastic pharmacy technicians (8×); pharmacy technicians who were used to providing EAFD and/or EASD (7×); positive reactions of patients (5×); project materials (2×); computer reminder at the moment of first or second dispensing (2×); patients were informed before they visited the pharmacy by a general practitioner or by means of a letter (2×); pharmacist gave feedback to pharmacy technicians (1×); materials on a visible place (1×); EAFD and EASD fitted well into the regular work system (1×). Pharmacies which performed best in this study did not report obviously different barriers or facilitators than pharmacies which did not perform well.

### Effect of education at first and second dispensing

Table 5 shows the use of statins related to whether patients received education at first or second dispensing as recommended by the study protocol. In the group of patients who did receive EAFD as recommended, significantly more patients received a second statin prescription: 93.9% versus 83.2% in the group of patients who did not receive EAFD as recommended (*P *= 0.028). The difference was no longer significant after 6 months: 80.7% of the patients who did receive EAFD as recommended used the statin for at least 6 months versus 77.0% of the patients who did not receive EAFD as recommended (*P *= 0.867). A non-significant difference was also found between patients who did versus patients who did not receive EASD: 91.2% of the patients in the first group still used the statin after 6 months, versus 83.2% of the patients in the second group (*P *= 0.216).

**Table 5:** Use of statins related to receiving education at dispensing

	Yes	No	*P*-value
Number of patients who received a second statin prescription (%) related to whether they had received EAFD as recommended (yes/no)	92/98 (93.9)	643/773 (83.2)	0.028

Number of patients who still used a statin 6 months after first dispensing (%) related to whether they had received EAFD as recommended (yes/no) ^a^	71/88 (80.7)	435/565 (77.0)	0.867

Number of patients with a second statin prescription who still used a statin 6 months after first dispensing (%) related to whether they had received EASD (yes/no) ^a^	73/80 (91.2)	421/506 (83.2)	0.216

^a ^Data from patients with at least 6 months follow up after first dispensing.

## Discussion

Only half of all pharmacies provided EAFD to at least one patient and in the pharmacies which did, less than one third of the patients received EAFD on average. Apparently, the intensive implementation program was not able to overcome the main barriers or to enhance factors needed for providing EAFD and EASD to new statin users. Pharmacies in the intervention group did not provide more education to patients at first or second dispensing of statins compared to the pharmacies in the control group. A review of 36 studies on patient education on medication [3] indicated that 29-87% of pharmacists or other pharmacy staff counselled 40-69% of all patients with a prescription for new medication. So, the finding that half of the pharmacies provided patient education in our study is similar to previous findings, but the percentage of new statin users receiving EAFD in our study was low compared to other research.

None of the characteristics which were related to the pharmacy's care providing function in previous studies [12,19] or of the other factors we investigated was related to providing EAFD. It is remarkable that being used to providing EAFD as reported by the pharmacist at the start of the study, did not influence the frequency of EAFD provided in this study. Nevertheless, seven project assistants from the experimental group spontaneously reported it as a facilitator for implementation. Possibly, the extent to which EAFD was provided in the participating pharmacies before we started the study, was of importance. For patient education on the negative effects of benzodiazepines, Ten Wolde et al. [20] reported that pharmacists' intention to provide education was higher if they expected more positive outcomes and if they experienced stronger social pressure to provide education. It is very well possible that pharmacists' intention to provide EAFD and EASD in our study may have influenced the implementation. A substantial number of pharmacies started late or not at all, with EAFD and EASD, because pharmacists waited for GPs to have checked the selected patients for an actual statin indication. Possibly, some pharmacists were not only rather passive in retrieving the lists from general practitioners, but in managing the team of pharmacy technicians as well. It is remarkable that pharmacies in which project assistants reported very positively about enthusiastic pharmacy technicians as a facilitator for implementation, did not necessarily provide more EAFD and EASD. Whether the intensive implementation strategy should have been directed not mainly at the project assistants, but also more at the pharmacists, will be a topic for further research.

In the pharmacies which started late with EAFD and EASD, patients might have received a statin before the pharmacy had started providing education. If we had only taken into account the patients who received a statin after the pharmacies had started with EAFD, than we would have found that more than one third of them did receive EAFD, which is still a low percentage. The low number of first statin prescriptions may also have contributed to the limited provision of EAFD and EASD in both groups, because enough exposure is needed for pharmacy technicians to become skilled in providing a new service. However, in most pharmacies the pharmacy technicians were already used to providing patient education at first dispensing of (some types of) medication. Finally, the measurement of the provision of EAFD and EASD by means of point of care checklists that were filled in manually may have caused underreporting of EAFD and EASD.

Although the intensive implementation program did not succeed in a better implementation of EAFD and EASD, it did have some effect on two elements of EAFD and EASD. Pharmacies in the experimental group appear to have chosen to focus on three aspects of EAFD, even if the patient did not fetch the medication himself/herself: read out loud the patient label with information about correct use; the indication for the statin; and the importance of its long-term use. This largely complies with the recommendation in the protocol for EAFD, which was also a subject in the second interactive educational meeting as a part of the intensive implementation strategy. Additionally, in the pharmacies who implemented EAFD and EASD, significantly more patients in the experimental group received EASD compared to patients in the control group. Experimental pharmacies provided EASD as frequently as they provided EAFD.

At the time this study was designed, providing EAFD and EASD was expected to improve medication adherence and persistence, even though the evidence for that was still lacking. This study was not designed to examine the effect of EAFD and EASD on statin persistence, so the results - which indicate a possible positive effect of EAFD on the short term - should be interpreted with caution. Possibly, there were differences in the quality of patient education between the control group and the experimental group, or differences in the education GPs had already given to their patients which could have attributed to the improved adherence in the experimental group. About 15% of the new statin users did not receive a second prescription, which indicates that use of statins rapidly decreases in an early stage, with a gradually smaller decrease on the longer term. This is in line with previous research [6]. In the meanwhile, several studies have been performed to investigate the effect of patient education on patients' adherence and persistence with statins. In general, interventions for improving adherence with chronic medication that have been shown to be effective are complex and do not lead to large improvements in adherence [21]. Studies which were published more recently have shown that simple interventions have not been able to increase patients' adherence with statins [22] or with other medicines for chronic use, among others lipid modifying agents [23]. A more extensive form of patient education, which existed of brief counselling by a physician followed by patient education mailings, has been shown to increase adherence with statins after four months [24]. Another intensive program has been shown to improve statin taking for at least one and a half year after cessation of the program [25], whereas another intensive program has been shown only to improve lipid levels in new statin users at three and six months after the start of the program, but not after one year [26]. Note that all residents in The Netherlands have an obligatory health insurance, which covers most medication. At the time of the study, there was no policy excess yet. So, out-of-pocket cost will not have hampered medication adherence in this study.

The randomized design was a strength of our study, although the statistical power was not focused on the outcomes reported here. As the pharmacies did not include the expected number of new statin users the number of patients included in this second part of the study remained low. It is possible that the intensive implementation program would have been more effective if more GPs would have cooperated actively in the first part of the trial and if more patients would have received a first statin prescription. Another weakness included the manual registration of EAFD and EASD by the pharmacy technicians. Occasionally they may have forgotten to complete the checklists, which may have led to underreporting of EAFD and EASD. On the other hand, manual registration at each first or second dispensing had the advantage that it was an objective measure and that recall bias could be avoided. By only using the checklists, we did not have any information on the quality of education provided.

## Conclusions

The intensive implementation program has not been able to increase the frequency of patient education provided at first and second dispensing of statins in community pharmacies, but it did accomplish that more patients received EASD in the pharmacies that implemented EASD. It remains unclear which factors contribute to successful implementation of EAFD and EASD. Although this study was not designed to investigate the effect of EAFD and EASD on statin adherence and persistence, the results indicate a possible positive effect on short term.

Because patient education is a basic element of pharmaceutical care, more efforts are required to implement EAFD and EASD on a large scale. Also, more research is needed to evaluate which factors influence their implementation and to develop a more effective implementation strategy. The effect of EAFD and EASD on patients' persistence with medication should be evaluated as well.

## Competing interests

The authors declare that they have no competing interests.

## Authors' contributions

CvdS participated in the design of the study, in the acquisition, analysis and interpretation of the data and drafted the manuscript. MW and PdS participated in the design of the study, in the interpretation of the data and were involved in critically revising the manuscript. All authors read and approved the final manuscript.

## Appendix

Attitude was measured using the construct developed by Muijrers et al [19]:

1. Pharmacists are the major medication experts in primary care.

2. The pharmacist should play a very important role in the care for patients who are actively using drugs.

3. Pharmacists greatly influence the use of medication.

4. The pharmacist should play a very important role in the drug prescription policy of the general practitioner.

5. Being a pharmacist, I hardly have any influence on the drug prescription policies of the general practitioners in my area of care.

6. To a major extent, the pharmacist is co-responsible for the drug therapy of his pharmacy's patients.

7. If a general practitioner is not sure about which drug therapy is most suitable for a given patient, the general practitioner should contact the pharmacist for advice.

8. When trying to make agreements within the PTAM group, the input of the pharmacist is essential.

9. The pharmacist should contribute to the PTAM agenda by supplying information about the prescription patterns observed in his/her pharmacy.

10. It is the task of the pharmacist to provide feedback to general practitioners with regard to their prescription figures.

11. The only responsibility of the pharmacist is to distribute drugs (i.e. providing the drug plus a brief instruction to the patient on how to use it, as well as monitoring potentially harmful interactions with other drugs).

12. The pharmacist should be aware of all the diagnosed conditions of a given patient which may affect medication response.

13. The pharmacist should be allowed to provide a pharmacotherapeutic substitute (i.e. an analogous drug, rather than the prescribed drug, is provided to the patient).

14. The pharmacist should be authorized to provide repeat-medication independently of the general practitioner.

15. It is the responsibility of the pharmacist to document undesirable prescription patterns of general practitioners.

16. In her/his role as medication expert, the pharmacist is responsible for the consequences of providing irrational drug therapy.

The following statement was omitted from this construct, because this is everyday practice in the Netherlands.

17. The pharmacist should be allowed to provide a generic drug instead of a prescribed specialty drug.

## Pre-publication history

The pre-publication history for this paper can be accessed here:

http://www.biomedcentral.com/1472-6963/11/313/prepub
